# Unexpected turn of events: Completing residency in the COVID-19 era

**DOI:** 10.36834/cmej.70516

**Published:** 2020-09-23

**Authors:** Marlon Danilewitz, Anees Bahji

**Affiliations:** 1 Department of Psychiatry, University of British Columbia, British Columbia, Canada; 2 Department of Psychiatry, Queen’s University, Ontario, Canada; 3 Department of Public Health Sciences, Queen’s University, Ontario, Canada

COVID-19 has had a profound impact on all medical trainees; however, the pandemic has had a unique effect on final-year residents. Thus, a looming question remains—how will we mark our graduation from training both at a program and an individual level?

For many, the completion of residency marks the end of a long journey of post-secondary rife with sacrifices and rewards. It is also a symbolic reflection of the shift towards an independent practice, and for some, real adulthood. Hence, it is a moment characterized by both excitement and trepidation. Ultimately, this transition is a poignant moment for reflection in any medical professional’s life. Often, it is these moments, typically characterized by formal ceremonies, celebrations, informal conversations and farewells between graduates and their colleagues, families, and other supports, which have fallen by the wayside.

However, COVID-19 has disrupted many of these milestones and opportunities for reflection in profound ways. With the cancellation or virtual displacement of graduation ceremonies and other important social gatherings, it is not surprising that there is a certain similarity with mourning or bereavement even. We will retain memories of the hurried or even forgotten goodbyes amidst the sea of COVID-19 meetings and clinical demands long after the pandemic has resolved.

Beyond the shift in how we commemorate graduation from medical training, COVID-19 has also complicated the transition from residency to independent practice. The uncoupling of residency completion from licensing examinations has created a murky change for many. Current graduates find themselves in an unprecedented grey zone marked by a myriad of unforeseeable challenges related to the pandemic, and an uncertain future.

The roadblocks associated with examinations, licensing, fellowships, among other issues, have highlighted to graduates the defined interests, roles, and accountabilities of licensing and regulatory bodies. In particular, the decision of the Royal College and the College of Family Physicians to delay licensing examinations by several months has impacted trainees profoundly.^1^^,^^2^ Several functional changes implemented by the national physician licensing bodies have led to both an amended exam (e.g., offering a virtual format in an increased number of testing facilities) and reduced provincial licensing requirements (e.g., waiving of the oral exam prerequisite for the Spring 2020 cohort).^1^^,^^2^ These challenges around licensing have led some residents to delay starting to practice until after the exam, have created obstacles for those pursuing fellowships abroad and forced residents to seek work at different hospital/clinic settings, which are receptive to hiring physicians with restricted licenses. One poignant example of this has been for family medicine residents pursuing the added competence in emergency medicine, which exam has been cancelled for the current cycle.^2^ Similarly, Canadian physicians seeking to complete the American Board of Psychiatry and Neurology, have as a result of the delay of Canadian licensing exams, been barred from the US exam, preventing those individuals from working in the US in this area of practice.

The delay has perhaps been most acutely felt by residents who have had to plan family decisions and make personal sacrifices to prepare for the exam. In turn, the delay of examinations has necessitated the creativity and flexibility of provincial colleges, with some issuing temporary restricted licenses of practice for would-be qualified physicians.^3^^,^^4^ For provinces like Ontario that have issued limited practice permits, would-be qualified physicians have now had to find supervision, which has perhaps been even more complicated for people who had planned to pursue independent practice or locums.^3^ The crystallization of this reality has led residents to take on greater self-advocacy, culminating in various discussions with the different stakeholders in both informal and formal settings.

The disruption induced by COVID-19 has provided trainees with a unique opportunity to reflect on the practice of medicine. Moreover, graduates continue to grapple with the interplay between individual and societal interests. Finding a balance between advocacy and acceptance will be the final hurdle that the current cohort must surpass before graduating, while also honouring themselves for completing the task of their medical training.
